# Successful Treatment of *Lactobacillus rhamnosus* Endocarditis With Intravenous Daptomycin: A Case Report and Literature Review

**DOI:** 10.1155/crdi/9295802

**Published:** 2026-01-26

**Authors:** Mehboob A. Rehan, J. Todd Bagwell, S. Blake Wachter, Sandeep Singh Jubbal

**Affiliations:** ^1^ Department of Medicine, Division of Infectious Diseases, Our Lady of Lordes Regional Medical Center, Lafayette, Louisiana, USA; ^2^ Department of Medicine, Eastern Idaho Regional Medical Center, Idaho Falls, Idaho, USA; ^3^ Department of Medicine, Division of Infectious Diseases University of Massachusetts Chan Medical School, Worcester, Massachusetts, USA

## Abstract

Lactobacilli are Gram‐positive, facultatively aerobic, rod‐shaped bacteria that are a normal part of the human microbiota and rarely cause infections in immunocompetent hosts. *Lactobacillus casei* and *Lactobacillus rhamnosus* are the most common human pathogens within this genus and are also frequently used in probiotics. Both species are inherently resistant to vancomycin, and penicillin—sometimes in combination with aminoglycosides—remains the primary treatment for infections caused by these organisms. Management becomes particularly challenging in patients with severe penicillin allergies, such as anaphylaxis. Although daptomycin has demonstrated in vitro activity against these pathogens, clinical data on its use are limited. Here, we present a case of *Lactobacillus rhamnosus* endocarditis successfully treated with 6 weeks of intravenous daptomycin.

## 1. Introduction

*Lactobacillus* genus was first described in 1901 by Beijerink and now consists of over 200 species [1, 2]. *Lactobacillus* is rod‐shaped, Gram‐positive, catalase‐negative, nonspore forming facultative aerobic bacteria [1, 3, 4]. They are part of the normal human flora found in the mouth, gastrointestinal (GI), and female genital tract [2–5]. *Lactobacilli* are uncommon human pathogens and are frequently considered contaminants if grown in blood cultures. They have increasingly been identified as a cause of bacteremia, infective endocarditis (IE), and intra‐abdominal abscesses including those of the liver and spleen. They are also identified in dental caries, urinary tract infections, and meningitis, especially in patients who are immunocompromised [4]. *Lactobacillus rhamnosus (L. rhamnosus*), *Lactobacillus casei* (*L. casei*), and *Lactobacillus paracasei (L. paracasei)* form the *L. casei* group. They are closely related and are the major components of probiotics. The two most common subspecies identified in human infections due to *Lactobacillus* are *L. casei* and *L. rhamnosus*. Given its use as probiotics [6], some authorities recommend caution when prescribing pro‐ or prebiotics to patients who are immunocompromised. Traditionally, penicillin is considered a treatment of choice for *Lactobacillus* bacteremia and *Lactobacillus* endocarditis. Sometimes an aminoglycoside is combined with penicillin due to their synergistic action against these organisms [6, 13]. Daptomycin, a lipopeptide antibiotic, has shown in vitro activity against *Lactobacillus* species; however, its clinical use and efficacy for *Lactobacillus* bacteremia and infection have only been reported in a few case reports in recent years [8, 24].

## 2. Case Report

A 68‐year‐old man with a history of mitral valve regurgitation, gastroesophageal reflux disease, Barrett’s esophagus, and chronic obstructive pulmonary disease (COPD) on long‐term prednisone therapy (20 mg daily) and baseline supplemental oxygen (2 L) was referred from an outpatient clinic after blood cultures grew Lactobacillus species in all four bottles. He initially presented to his outpatient provider with a one‐month history of intermittent episodes of transient left‐eye blindness lasting seconds to minutes, with progressively increasing frequency and duration. Associated symptoms included subjective fevers, chills, worsening dyspnea from baseline, fatigue, and unintentional weight loss of approximately 5–8 pounds over four weeks. During the initial outpatient evaluation, blood cultures were obtained and returned positive for Lactobacillus species in all four bottles. An outpatient transthoracic echocardiogram revealed a 15–20‐mm vegetation on the mitral valve with severe mitral regurgitation, without evidence of acute congestive heart failure, likely reflecting pre‐existing compensated valvular disease. No perivalvular abscess was identified. Species identification confirmed *Lactobacillus rhamnosus* in all four bottles by Bruker MALDI‐TOF. Repeat blood cultures obtained on Day 2 demonstrated persistent growth of *L. rhamnosus*. Antimicrobial susceptibility testing performed using the microbroth dilution method demonstrated resistance to vancomycin and susceptibility to daptomycin (MIC 0.5 μg/mL), penicillin (MIC 1 μg/mL), and clindamycin (MIC 0.06 μg/mL). Gentamicin susceptibility was not reported by the laboratory, and synergy testing was not pursued given the patient’s history of a life‐threatening penicillin allergy with prior respiratory distress and angioedema, which precluded the use of penicillin‐based therapy. Following admission, additional history was obtained. The patient denied recent probiotic use, dental procedures, illicit drug or alcohol use, or excessive dairy consumption. He reported remote occupational exposure, having worked in a cheese factory approximately ten years earlier. He also had a history of gastric bypass surgery performed several years prior for a benign gastric tumor but denied any recent gastrointestinal procedures, including endoscopy or colonoscopy. His diagnosis of mitral valve regurgitation had been established five years prior to presentation. The patient was admitted with a diagnosis of *L. rhamnosus* mitral valve infective endocarditis. On initial physical examination, he appeared chronically ill but was afebrile and hemodynamically stable, with an increased oxygen requirement via nasal cannula from 2 to 4 L. There were no signs of volume overload or impending heart failure, including normal jugular venous pressure and absence of peripheral edema or an S3 gallop. Cardiac examination revealed a Grade 3/6 holosystolic murmur best heard at the apex. Initial laboratory studies were notable for mild leukocytosis with neutrophilic predominance, acute kidney injury (creatinine 1.4 mg/dL), and mild transaminitis. At the time of admission to the hospital, two additional sets of blood cultures were obtained, and intravenous daptomycin was initiated at a dose of 10 mg/kg/day due to the patient’s severe penicillin allergy. Blood cultures obtained at admission (Day 4) remained positive for *L. rhamnosus* in all four bottles. Clearance of bacteremia was achieved by Day 6, with subsequent blood cultures on Days 8 and 10 remaining negative and accompanied by marked clinical improvement following initiation of daptomycin therapy. Magnetic resonance imaging of the brain revealed acute and chronic microembolic infarcts involving the right cerebellar hemisphere, left medial temporal lobe, left parietal lobe, and bilateral frontal lobes. Computed tomography angiography of the head and neck showed no evidence of carotid artery stenosis. The mitral valve vegetation was presumed to be the source of these embolic phenomena. The patient met two major criteria (persistently positive blood cultures and an oscillating intracardiac vegetation on echocardiography) and three minor criteria (fever, predisposing valvular heart disease, and vascular phenomena in the form of cerebral microemboli) of the modified Duke criteria, confirming a diagnosis of definitive infective endocarditis. The patient underwent surgical mitral valve replacement on Hospital Day 15. His postoperative course was complicated by bilateral iatrogenic pneumothoraces, pneumomediastinum, and new‐onset atrial fibrillation, all of which were managed successfully. He was subsequently discharged home in stable condition with a planned six‐week course of intravenous daptomycin.

## 3. Discussion

IE was first described in 1885 by Sir William Osler [7]. The annual incidence of IE is reported to be around 3.6 per 100,000 population every year. The incidence of *Lactobacillus* endocarditis is extremely rare, and only 0.5% of all endocarditis cases are due to *Lactobacillus* [9]. Traditionally, *lactobacilli* are considered contaminants in blood cultures unless growth is persistent on repeated blood cultures, or all four bottles show growth of lactobacillus species in a patient with clinically compatible signs and symptoms of sepsis. Generally, *Lactobacilli* are extremely rare to grow in blood cultures. In one study, *Lactobacillus* grew in only 0.1% of all blood cultures over a 5‐year period, and 66% of these isolates were identified as *L. rhamnosus* bacteremia in immunosuppressed patients [10].

Various studies have reported high mortality in endocarditis caused by anaerobic organisms, ranging between 21% and 43% [11]. However, although *Lactobacillus* endocarditis is rare, it carries an even higher mortality risk than these anaerobic pathogens. Cannon et al., in their retrospective review of more than 200 cases of *Lactobacillus* infection, reported an overall mortality of 30% [12]. In the same review, 63% of patients with *Lactobacillus* endocarditis had structural heart disease, including prosthetic and native valvular diseases [12]. Another retrospective study on *Lactobacillus* bacteremia reported even higher mortality, with case mortality of 28% at day and 28% and 55.1% at 1 year [13]. In a retrospective analysis of 89 patients with *Lactobacillus* bacteremia, Salminen et al. (2004) documented mortality rates of 26% at 1 month and 48% at 1 year.

*Lactobacillus* almost always infects seriously ill patients with underlying immunosuppression, structural heart disease (especially valvular heart disease), and/or malignancy. These underlying conditions appear to be the major risk factors for mortality in systemic *Lactobacillus* infection. Thus, occurrence of *Lactobacillus* bacteremia should necessitate further work‐up to investigate undiagnosed immunosuppression, presence of structural heart disease, or underlying occult malignancy.

Several risk factors for *Lactobacillus* bacteremia and endocarditis have been reported in medical literature [5, 9, 11–15] including immunosuppression (current or chronic immunosuppressive therapy, acquired immunodeficiency syndrome [AIDS], or chemotherapy within 2 weeks of bacteremia), active cancer/neoplasm (defined as active treatment or in remission within 6 months), valvular heart disease including involvement of prosthetic heart valves, central venous line implanted before bacteremia, recent upper respiratory or GI tract surgery or upper GI endoscopy, prior prolonged hospitalization, prolonged antibiotic therapy, and diabetes mellitus. Excessive dairy product consumption, probiotic use, and poor dentition have also been reported as potential risk factors.

It is important to note that *Lactobacillus rhamnosus* is intrinsically resistant to vancomycin. The susceptibility of *L. rhamnosus* to glycopeptide antibiotics is influenced by its glucose‐fermentation pattern. Lactobacilli, a group of lactic acid bacteria (LAB), ferment carbohydrates to generate ATP via substrate‐level phosphorylation. LAB are classified as either homofermentative or heterofermentative: the former uses the Embden–Meyerhof pathway to convert glucose to pyruvate, whereas the latter relies on the phosphoketolase pathway to produce ethanol, CO_2_, and lactate. *Lactobacillus rhamnosus* is heterofermentative and naturally resistant to vancomycin, whereas *Lactobacillus acidophilus* is homofermentative and therefore susceptible to vancomycin. Almost all heterofermentative LAB (e.g., *Lactobacillus*, *Leuconostoc*, *Weissella*, and *Oenococcus*) are inherently resistant to vancomycin due to D‐Ala–D‐Lac cell wall precursors [25]. The reason is unclear but may relate to their metabolism. Consequently, vancomycin is usually avoided in *Lactobacillus* bacteremia, where *L. rhamnosus* and *L. casei* predominate. In contrast to vancomycin, a glycopeptide, daptomycin is a lipopeptide antibiotic. Discovered in the early 1980s, it was approved by the FDA in November 2003 for skin and soft tissue infections caused by Gram‐positive pathogens at 4 mg/kg/day, and in 2006 for *Staphylococcus aureus* bacteremia at 6 mg/kg/day. The lipophilic end of daptomycin inserts into the bacterial cell membrane, causing depolarization, potassium influx, and bacterial cell death [16]. Steenberg et al. (2005) reported that daptomycin exhibits a minimum inhibitory concentration (MIC) range of < 0.03–32 μg/mL against 37 *Lactobacillus* strains [16]. King and Phillips demonstrated the in vitro activity of daptomycin against various Gram‐positive bacteria, including vancomycin‐resistant enterococci, reporting MICs for these isolates—including *Lactobacillus* spp.—ranging from 0.03 to 2 μg/mL on Mueller–Hinton and supplemented isotonic agar (50 mg/L Ca^2+^) [17]. The MIC of daptomycin for *L. rhamnosus* in our patient was 0.5 μg/mL, making it a suitable choice, particularly given the patient’s history of severe penicillin‐induced anaphylaxis. It was evident that our patient experienced an immunoglobulin E (IgE)‐mediated type I hypersensitivity reaction to penicillin, which can be life‐threatening. Intravenous penicillin desensitization protocols typically require 9–12 h or longer to complete [18]. Given the excellent clinical response and rapid clearance of bacteremia, penicillin desensitization was deemed unnecessary. IE can lead to a range of neurological complications, including ischemic or hemorrhagic stroke, infected intracranial aneurysm, meningitis, brain abscess, spinal epidural abscess, encephalopathy, and seizures. Stroke is the most common neurological complication of IE, clinically manifesting in 20%–40% of patients, while incidental ischemic lesions are detected on neuroimaging in 30%–40% of patients without neurological deficits. Infected intracranial aneurysm, brain abscess, and meningitis are rare, with reported incidences of 1%–2%, although higher rates are observed in cases caused by *Staphylococcus aureus* [19]. Our patient showed evidence of septic emboli to the brain on neuroimaging but had no nuchal rigidity, focal neurological deficits, or altered mental status. Traditionally, daptomycin is not considered effective at penetrating the blood‐brain barrier or achieving therapeutic cerebrospinal fluid (CSF) concentrations due to high protein binding and is therefore generally avoided in central nervous system infections. Various animal studies have demonstrated that daptomycin achieves CSF penetration of up to 2% in noninflamed meninges, increasing to approximately 6% in the presence of meningeal inflammation [20]. In their prospective observational study of nine patients with meningitis, Piva et al. [21] reported a mean daptomycin CSF penetration of 0.45%, with a peak concentration of 0.21 mg/L at a dose of 10 mg/kg/day. In contrast, the majority of animal and clinical studies have reported daptomycin CSF penetration of 4%–7%, with peak CSF concentrations ranging from 0.52 mg/L in humans at a daily dose of 9 mg/kg to 4.5 mg/L in rabbits receiving 15 mg per day [22, 23, 26]. Even if we assume that our patient developed meningitis secondary to *Lactobacillus rhamnosus* septic emboli, a CSF peak concentration of at least 0.52 mg/L could still have been achieved, which approximates the MIC and is considered therapeutic.

Our patient was successfully treated with surgical valve replacement and intravenous daptomycin at a dose of 10 mg/kg/day for 6 weeks, resulting in complete recovery. Creatine kinase levels were monitored weekly and remained within the normal range throughout treatment. In a recently published case report, *Lactobacillus rhamnosus* bacteremia and endocarditis without evidence of peripheral emboli were successfully treated with daptomycin at a dose of 8 mg/kg/day [24]. In our patient, however, we opted for 10 mg/kg/day due to central nervous system involvement and following infectious diseases recommendations to use higher doses for bacteremia and endocarditis.

## 4. Conclusion

Our case highlights the potential role of daptomycin in the treatment of *Lactobacillus* bacteremia and IE, particularly in patients with severe, life‐threatening penicillin allergies. It adds to the limited number of published reports demonstrating successful treatment of *Lactobacillus rhamnosus* bacteremia and endocarditis with daptomycin in clinical practice. Furthermore, our case illustrates that daptomycin can effectively eradicate infection in the central nervous system despite traditional concerns regarding its CNS penetration. More studies are needed to further elucidate daptomycin’s efficacy against vancomycin‐resistant *Lactobacillus* species and its CNS penetration.

## Ethics Statement

The authors declare that this work complies with reporting quality, formatting, and reproducibility guidelines established by the EQUATOR Network. The authors also confirm that this clinical investigation did not require review by an Institutional Review Board or Ethics Committee. Formal consent was obtained from the patient for this report.

## Consent

The authors certify that they have obtained all appropriate patient consent forms. In these forms, the patient has provided consent for his clinical information and images to be reported in the journal. The patient understands that his name and initials will not be published and that all efforts will be made to protect his identity.

## Disclosure

This research was supported (in whole or in part) by HCA Healthcare and/or an HCA Healthcare affiliated entity. The views expressed in this publication represent those of the author(s) and do not necessarily represent the official views of HCA Healthcare or any of its affiliated entities.

## Conflicts of Interest

The authors declare no conflicts of interest.

## Funding

No funding was received for this manuscript.

## Data Availability

Data sharing is not applicable to this article as no datasets were generated or analyzed during the current study.
